# Exact and Heuristic Methods for Network Completion for Time-Varying Genetic Networks

**DOI:** 10.1155/2014/684014

**Published:** 2014-03-09

**Authors:** Natsu Nakajima, Tatsuya Akutsu

**Affiliations:** Bioinformatics Center, Institute for Chemical Research, Kyoto University, Gokasho, Uji, Kyoto 611-0011, Japan

## Abstract

Robustness in biological networks can be regarded as an important feature of living systems. A system maintains its functions against internal and external perturbations, leading to topological changes in the network with varying delays. To understand the flexibility of biological networks, we propose a novel approach to analyze time-dependent networks, based on the framework of network completion, which aims to make the minimum amount of modifications to a given network so that the resulting network is most consistent with the observed data. We have developed a novel network completion method for time-varying networks by extending our previous method for the completion of stationary networks. In particular, we introduce a double dynamic programming technique to identify change time points and required modifications. Although this extended method allows us to guarantee the optimality of the solution, this method has relatively low computational efficiency. In order to resolve this difficulty, we developed a heuristic method for speeding up the calculation of minimum least squares errors. We demonstrate the effectiveness of our proposed methods through computational experiments using synthetic data and real microarray gene expression data. The results indicate that our methods exhibit good performance in terms of completing and inferring gene association networks with time-varying structures.

## 1. Introduction

Computational analysis of gene regulatory networks is an important topic in systems biology. A gene regulatory network is a collection of genes and their correlations and causal interactions. It is often represented as a directed graph in which the nodes correspond to genes and the edges correspond to regulatory relationships between two genes. Gene regulatory networks play important roles in cells. For example, gene regulatory networks maintain organisms through protein production, response to the external environment, and control of cell division processes. Therefore, deciphering gene regulatory network structures is important for understanding cellular systems, which might also be useful for the prediction of adverse effects of new drugs and the detection of target genes for the development of new drugs. In order to infer gene regulatory networks, various kinds of data have been used, such as gene expression profiles (particularly mRNA expression profiles), CHromatin ImmunoPrecipitation (ChIP)-chip data for transcription binding information, DNA-protein interaction data, and protein-protein interaction data [1–3]. However, many existing studies have focused on the use of gene expression profiles, because expression data from a large number of genes can be simultaneously observed due to developments in DNA microarray technology [1–3]. Various mathematical models and computational methods have been applied and/or developed to infer gene regulatory networks from gene expression profiles, which include Boolean networks [4, 5], Bayesian networks [6, 7], dynamic Bayesian networks [8], differential equations [9, 10], and graphical Gaussian models [11]. In Boolean networks, the state of each gene is simplified into 0 or 1 and the gene regulation rules are given as Boolean functions, where 0 and 1 mean that a gene is active (in high expression) and inactive (in low expression), respectively. In the most widely used Boolean network model, it is assumed that the states of genes change synchronously according to discrete time steps. In Bayesian networks, the states of genes are usually classified into discrete values and the gene regulation rules are given in the form of conditional probabilities. Although standard Bayesian networks can only handle static data and acyclic networks, dynamic Bayesian networks can handle time series data and cyclic networks. In differential equation models, the dynamics of gene expression are represented by a set of linear or nonlinear equations (one equation per gene). In graphical Gaussian models, partial correlations are used as a measure of independence of any two genes, by which direct interactions are distinguished from indirect interactions. For details of these models and methods, see review/comparison papers [1–3].

These network models assume that the topology of the network does not change through time, whereas the real gene regulatory network in the cell might dynamically change its structure depending on time, the effects of certain shocks, and so forth. Therefore, many reverse engineering tools have recently been proposed, which can reconstruct time-varying biological networks based on time-series gene expression data. Yoshida et al. [12] developed a dynamic linear model with Markov switching that represents change points in regimes that evolve according to a first-order Markov process. Fujita et al. [13] proposed a method based on the dynamic autoregressive model. This model extends the vector autoregression (VAR) model, which can be applied to the inference of nonlinear time-dependent biological correlations such as dynamic gene regulatory networks. Robinson and Hartemink [14] proposed a model called a nonstationary dynamic Bayesian network, based on dynamic Bayesian networks, which allows inference from data generated by nonstationary processes in a time-dependent manner. Lèbre et al. [15] also introduced the autoregressive time-varying (ARTIVA) algorithm for the analysis of time-varying network topologies from time course data, which is generated from different processes. This model adopts a combination of reversible jump Markov chain Monte Carlo (RJMCMC) and dynamic Bayesian networks (DBN), in which RJMCMC is used for the identification of change time points and the resulting networks, and DBN is used to represent causal interactions among genes. Thorne and Stumpf [8] presented a method to model the regulatory network structure between distinct segments with a set of hidden states by applying the hierarchical Dirichlet process hidden Markov model [16], including a potentially infinite number of states and a Bayesian network model for estimating relationships between genes. Rassol and Bouaynaya [17] presented a new method based on constrained and smoothed Kalman filtering, which is capable of estimating time-varying networks from time-series data, including unobserved and noisy measurements. The dynamics of genetic modules are represented as a linear-state space equation and the observability of linear time-varying systems is defined by imposing sparse constraints in Kalman filters. Ahmed et al. [18] proposed an algorithm called Tesla with machine learning, which can be cast in the form of a convex optimization problem. The basic assumption in this method is that networks at close time points do not have significant topological differences but have common edges with high probability; in contrast, networks at distant time points are markedly different. The regulatory networks are represented by Markov random fields at arbitrary time intervals.

As mentioned above, there have been many studies and attempts to analyze both time-independent and time-dependent networks from time-series expression data; however, gene regulatory systems in living organisms are so complicated that any mathematical model has limitations and there is not yet a standard or established method for inference, even for time-independent networks. One of the possible reasons is that there exists an insufficient number of high-quality time-series datasets to reconstruct the dynamic behavior of the network. In other words, it is difficult to reveal a correct or nearly correct network based on a small amount of data that includes some noise. Hence, in our recent study, we proposed a new approach for the analysis of time-independent networks, called network completion [19, 20], in which the minimum amount of modifications are made to a given network so that the resulting network is most consistent with the observed data. Similar concepts have been independently proposed [21–24]. In addition, network completion can be applied to inference of networks by starting with the null network.

In this paper, we present two novel methods for the completion and inference of time-varying networks using dynamic programming and least squares fitting (DPLSQ): DPLSQ-TV (DPLSQ-TV was presented in a preliminary version of this paper [25]; however, in this paper, more detailed computational experiments are performed and DPLSQ-HS is newly introduced) and DPLSQ-HS, where TV and HS stand for time varying and heuristics. DPLSQ-TV is an extension of DPLSQ [20] such that it can identify the time points at which the structure of the gene regulatory network changes. Since the additions and deletions of edges are basic modifications in network completion, we need to extend DPLSQ so that these operations can be performed at several time points. In DPLSQ-TV, these edges and time points are identified by a novel double dynamic programming method in which the inner loop is used to identify static network structures and the outer loop is used to determine change points. It is to be noted that a single dynamic programming (DP) method was used in our previous work on the completion and inference of time-independent networks [20], whereas a double DP method is employed here in order to cope with time-varying networks. Our proposed methods also allow us to find an optimal solution in polynomial time if the maximum indegree (i.e., the maximum number of input genes to a gene) is bounded by a constant. Although DPLSQ-TV is guaranteed to find an optimal solution in polynomial time, the degree of the polynomial is not low, which prevents the method from being applied to the completion of large networks. Therefore, we further propose a heuristic method, called DPLSQ-HS, to speed up the calculation of the minimum least squares error by applying restriction constraints that limit the number of combinations of incoming nodes.

We evaluate the efficiency of our methods through computational experiments using synthetic data and microarray gene expression data from the life cycle of* D. melanogaster* and the cell cycle of* S. cerevisiae.* We also demonstrate the effectiveness of the proposed methods by comparing our results with those of ARTIVA [15].

## 2. Method

In this section, we present DPLSQ-TV, a DP-based method for the completion of a time-varying network. We assume that there exist *m* time points (1,2,…, *m*), which are divided into *B* + 1 intervals: [1,…, *c*_1_ − 1], [*c*_1_,…, *c*_2_ − 1],…, [*c*_*B*_,…, *m*], where *B* indicates the number of change points. A different network is associated with each interval. We assume that the set of genes does not change; therefore, only the edge set changes according to the time interval. Let *V* = {*v*_1_,…, *v*_*n*_} be the set of genes. Let *E* be the initial set of directed edges (i.e., initial set of gene regulation relationships), and let *E*_0_, *E*_1_,…, *E*_*B*_ be the sets of directed edges (i.e., the output), where *E*_*i*_ denotes the edge set for the *i*th interval.

**Figure 1:**
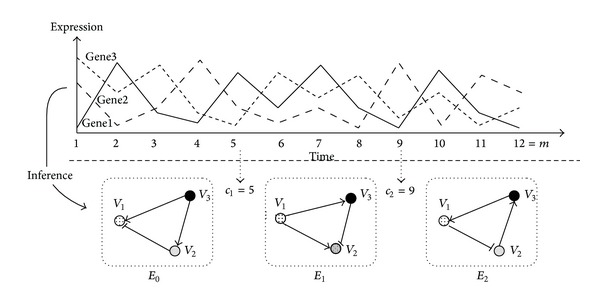
Inference (i.e., completion starting with the null network) of time varying structure of a genetic network. This example corresponds to the case of *N* = 1, *n* = 3, *B* = 2, and *m* = 12. The change points are *c*_1_ = 5 and *c*_2_ = 9.

Then, the problem is defined as follows: given an initial network *G*(*V*, *E*) consisting of *n* genes, *N* time series datasets, each of which consists of *m* time points for *n* genes and the positive integers *h*, *k*, and *B*, infer *B* change points (i.e., *c*_1_, *c*_2_,…, *c*_*B*_) and complete the initial network *G*(*V*, *E*) by adding *k* edges and deleting *h* edges in total such that the total least-squares error is minimized. This results in the set of edges *E*_0_, *E*_1,_,…, *E*_*B*_ at the corresponding time intervals (see Figure 1). It is to be noted that if we start with an empty set of edges (i.e., *E* = *∅*), the problem corresponds to the inference of a time-varying network.

### 2.1. Model of Differential Equation and Estimation of Parameters

**Figure 2:**
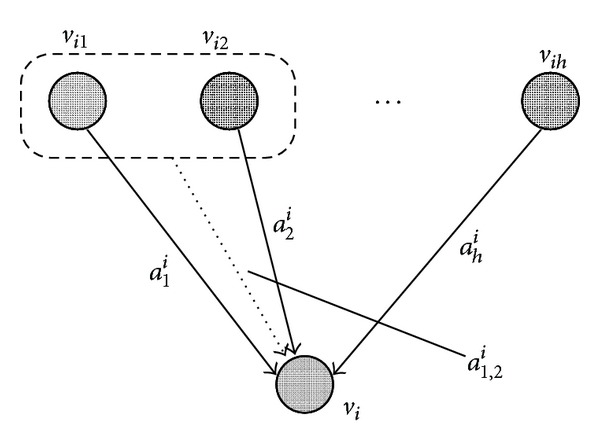
Dynamics model for a node.

We assume that the dynamics of each node *v*_*i*_ are determined by the following differential equation:

(1) $$\begin{array}{c} \frac{dx_{i}}{dt}=a_{0}^{i}+\overset{h}{\underset{j=1}{\sum }}a_{j}^{i}x_{i_{j}}+\underset{j<k}{\sum }a_{j,k}^{i}x_{i_{j}}x_{i_{k}}+b^{i}\omega , \end{array}$$

where *x*_*i*_ corresponds to the expression value of node *v*_*i*_, *ω* denotes random noise, and *v*_*i*_1__,…, *v*_*i*_*h*__ are incoming nodes to *v*_*i*_. The second and third terms of the right-hand side of the equation represent the linear and nonlinear effects to node *v*_*i*_, respectively (see Figure 2), where a positive value for *a*_*j*_^*i*^ or *a*_*j*,*k*_^*i*^ corresponds to an activation effect, and a negative value for *a*_*j*_^*i*^ or *a*_*j*,*k*_^*i*^ corresponds to an inhibition effect. This model is an extension of the linear differential equation model [3]. It is also a variant of the recurrent neural network model [27], although the sigmoid function is replaced here by an identify function and nonlinear terms representing cooperating regulations are added instead.

In practice, we replace the derivative of (1) by the difference and ignore the noise term as follows:

(2) xi(t+1)  =xi(t)+Δt(a0i+∑j=1hajixij(t)+∑j<kaj,kixij(t)xik(t)),

where Δ*t* denotes the unit time. This kind of discretization is also employed for linear and recurrent neural network models [3, 27].

In our previous method DPLSQ [20], we assume that time series data *y*_*i*_(*t*)s, which correspond to *x*_*i*_(*t*)s in (2), are given for *t* = 0,1,…, *m*, where we distinguish an observed expression value *y*_*i*_(*t*) from an expression value *x*_*i*_(*t*) in the mathematical model equation (2). Then, the parameters *a*_*j*_^*i*^s and *a*_*j*,*k*_^*i*^s are estimated from these time series data by minimizing the following objective function (i.e., the sum of the least squares errors) for each node *v*_*i*_:

(3) ∑t=1m|yi(t+1)  −[yi(t)+Δt(a0i+∑j=1hajiyij(t)+∑j<kaj,kiyij(t)yik(t))]|2.

It should be noted that *y*_*i*_(*t*) is the observed expression value of gene *v*_*i*_ at time *t*, and *v*_*i*_1__, *v*_*i*_2__,…, *v*_*i*_*h*__ are tentative incoming nodes to node *v*_*i*_. Incoming nodes to each node are determined so that the sum of these values for all nodes is minimized under the constraint that the total number of edges is equal to the specified number. In order to minimize the sum of least squares errors for all genes along with determining the incoming nodes and corresponding parameters, DP is applied. Readers are referred to [20] for the details of DPLSQ.

### 2.2. Completion by Addition of Edges

In this subsection, we present our proposed method for network completion of time-varying networks by the addition of edges and extend this to a general case (i.e., network completion by the addition and deletion of edges) in the following subsection. For simplicity, we assume *N* = 1, where we can extend the method to the case of *N* > 1 by changing the definition of *S*_{*v*_*j*_1__,*v*_*j*_2__,…,*v*_*j*_*d*__}_^*i*^[*p*, *q*] only.

We assume that the set of nodes (i.e., the set of genes) *V* and the set of initial edges *E* are given. Let the current set of incoming nodes to *v*_*i*_ be {*v*_*i*_1__,…, *v*_*i*_*d*__}. We define the least squares error for *v*_*i*_ during the time period between *p* and *q* as

(4) S{vi1,vi2,…,vid}i[p,q]=min⁡a0i,aji,aj,ki∑t=pq−1|yi(t+1)−[yi(t)+Δt×(a0i+∑j=1dajiyij(t)+∑j<kaj,kiyij(t)yik(t))]|2,

where *y*_*i*_(*t*) denotes the observed expression value of gene *v*_*i*_ at time *t*. The parameters (i.e., *a*_0_^*i*^, *a*_*j*_^*i*^, *a*_*j*,*k*_^*i*^) needed to attain this minimum value can be computed by a standard least squares fitting method.

Because network completion is considered to involve the addition of edges, let *e* ^−^(*v*_*i*_) = {*v*_*j*_1__,…, *v*_*j*_*d*__} be the set of initial incoming nodes to *v*_*i*_. Let *σ*_*k*_*j*_,*j*_^+^[*p*, *q*] denote the minimum least squares error when adding *k*_*j*_ edges to the *j*th node during the time from *p* to *q*, which is formally defined as

(5) $$\begin{array}{c} \sigma_{k_{j},j}^{+}\left[p,q\right]=\underset{j_{1},j_{2},\ldots ,j_{k_{j}}}{\min}\left\{S_{e^{-}\left(v_{j}\right)\cup \{v_{j_{1}},v_{j_{2}},\ldots ,v_{j_{k_{j}}}\}}^{j}\left[p,q\right]\right\}, \end{array}$$

where each *v*_*j*_*l*__ must be selected from *V* − *v*_*j*_ − *e* ^−^(*v*_*j*_). In order to avoid combinatorial explosion, we constrain the maximum *k*_*j*_ to be a small constant, *K*, and let *σ*_*k*_*j*_,*j*_^+^[*p*, *q*] = +*∞*, for *k*_*j*_ > *K* or *k*_*j*_ + |*e* ^−^(*v*_*j*_) | ≥*n*.

Then, the problem is stated as

(6) min⁡c1<c2<⋯<cBk0+k1+⋯+kB=k⁡{∑i=0Bmin⁡k1+k2+⋯+kn=ki[∑j=1nσkj,j+[ci,ci+1−1]]},

where *c*_0_ = 1 and *c*_*B*+1_ − 1 = *m*.

Here, we define *D* ^+^[*k*, *i*, *p*, *q*] as

(7) $$\begin{array}{c} D^{+}\left[k,i,p,q\right]\;=\;\underset{k_{1}+k_{2}+\cdots +k_{i}=k}{\min}\left\{\overset{i}{\underset{j=1}{\sum }}\sigma_{k_{j},j}^{+}\left[p,q\right]\right\}. \end{array}$$

The entries of *D* ^+^[*k*, *j*, *p*, *q*] can be computed by the following DP algorithm:

(8) $$\begin{array}{c} D^{+}\left[k,1,p,q\right]=\sigma_{k,1}^{+}\left[p,q\right],\\ D^{+}\left[k,j+1,p,q\right]=\underset{k^{\prime }+k^{\prime \prime }=k}{\min}\left\{D^{+}\left[k^{\prime },j\right]+\sigma_{k^{\prime \prime },j+1}^{+}\left[p,q\right]\right\}. \end{array}$$

It is to be noted that *D* ^+^[*k*, *n*, *p*, *q*] is determined uniquely regardless of the ordering of nodes in the network. The correctness of this DP algorithm can be seen as follows:

(9) min⁡k1+k2+⋯+kn=k{∑j=1nσkj,j+[p,q]} =min⁡k′+k′′=k{min⁡k1+k2+⋯+kn−1=k′∑j=1n−1σkj,j+[p,q]+σk′′,n+[p,q]} =min⁡k′+k′′=k{D+[k′,n−1,p,q]+σk′′,n+[p,q]}.

Next, we define *E* ^+^[*k*, *b*, *q*] as

(10) E+[k,b,q]=min⁡c1<c2<⋯<cbk0+k1+⋯+kb=k⁡{∑i=0bmin⁡k1+k2+⋯+kn=ki[∑j=1nσkj,j+[ci,ci+1−1]]},

where *c*_*b*+1_ − 1 = *q*. *E* ^+^[*k*, *b*, *q*] can be computed by the following DP algorithm:

(11) E+[k,0,q]=D+[k,n,1,q],E+[k,b,q]  =min⁡p<qk′+k′′=k{D+[k′,n,p,q]+E+[k′′,b−1,p]}.

The introduction of *E* ^+^[*k*, *b*, *q*] and the corresponding DP procedure are the methodologically novel points of this work, compared with our previous work [20].

The correctness of this DP algorithm can be seen as follows:

(12) min⁡c1<c2<⋯<cbk0+k1+⋯+kb=k⁡{∑i=0bmin⁡k1+k2+⋯+kn=ki[∑j=1nσkj,j+[ci,ci+1−1]] }=min⁡cb−1<cbk′+k′′=k⁡{min⁡c1<⋯<cb−1k0+⋯+kb−1=k′{∑i=0b−1min⁡k1+⋯+kn=ki×[∑j=1nσkj,j+[ci,ci+1−1]]}+min⁡k1+⋯+kn=k′′[∑j=1nσkj,j+[p,q]]}=min⁡p<qk′+k′′=k{E+[k′,b−1,p]+D+[k′′,n,p,q]}.

### 2.3. Completion by Addition and Deletion of Edges

The above DP procedure can be modified for the deletion of edges and for the addition and deletion of edges as in DPLSQ [20]. Since the former case is a subcase of the latter one, we describe only the latter one (addition and deletion of edges) here.

Let *σ*_*h*_*j*_,*k*_*j*_,*j*_[*p*, *q*] denote the minimum least squares error for the time period between *p* and *q* when adding *k*_*j*_ edges to *e* ^−^(*v*_*j*_) and deleting *h*_*j*_ edges from *e* ^−^(*v*_*j*_), where the added and deleted edges must be disjointed. We constrain the maximum *k*_*j*_ and *h*_*j*_ to the small constants *K* and *H*. We let *σ*_*h*_*j*_,*k*_*j*_,*j*_[*p*, *q*] = +*∞* if *k*_*j*_ > *K*, *h*_*j*_ > *H*, *k*_*j*_ − *h*_*j*_ + |*e* ^−^(*v*_*j*_) | ≥*n*, or *k*_*j*_ − *h*_*j*_ + |*e* ^−^(*v*_*j*_) | <  0 hold. Then, the problem is stated as

(13) min⁡c1<c2<⋯<cBk0+k1+⋯+kB=kh0+h1+⋯+hB=h⁡{∑i=0B ‍min⁡k1+k2+⋯+kn=kih1+h2+⋯+hn=hi⁡[∑j=1nσhj,kj,j[ci,ci+1−1]]}.

Here, we define *D*[*h*, *k*, *i*, *p*, *q*] as

(14) D[h,k,i,p,q]=min⁡k1+k2+⋯+ki=kh1+h2+⋯+hi=h⁡{∑j=1iσhj,kj,j[p,q]}.

As in the previous subsection, *D*[*h*, *k*, *j*, *p*, *q*] can be computed by

(15) D[h,k,1,p,q]=σhj,kj,j[p,q],D[h,k,j+1,p,q]  =min⁡k′+k′′=kh′+h′′=h⁡{D[h′,k′,j,p,q]+σh′′,k′′,j+1[p,q]}.

Next, we define *E*[*h*, *k*, *b*, *q*] as

(16) E[h,k,b,q]=min⁡c1<c2<⋯<cbk0+k1+⋯+kb=kh0+h1+⋯+hb=h{∑i=0b min⁡k1+k2+⋯+kn=kih1+h2+⋯+hn=hi⁡[∑j=1nσhj,kj,j[ci,ci+1−1]]}.

*E*[*h*, *k*, *b*, *q*] can be computed by the following DP algorithm:

(17) E[h,k,0,q]=D[h,k,n,1,q],E[h,k,b,q]  =min⁡p<qk′+k′′=kh′+h′′=h⁡{D[h′,k′,n,p,q]+E[h′′,k′′,b−1,p]}.

### 2.4. Time Complexity Analysis

In this subsection, we analyze the time complexity of DPLSQ-TV. Since completion by the addition of edges and completion by the deletion of edges are special cases of completion by the addition and deletion of edges, we focus on completion by the addition and deletion of edges.

First, we analyze the time complexity required per least squares fitting. It is known that least squares fitting for a linear system can be done in *O*(*mp* ^2^ + *p* ^3^) time where *m* is the number of data points and *p* is the number of parameters. In our proposed method, we assume that the maximum indegree is bounded by a constant, and the numbers of addition and deletion edges in a given network are bounded by the constants *K* and *H*, respectively. In this case, the time complexity for least squares fitting can be estimated as *O*(*m*).

Next, we analyze the time complexity required for computing *σ*_*h*_*j*_,*k*_*j*_,*j*_[*p*, *q*]. The total time required to compute *σ*_*h*_*j*_,*k*_*j*_,*j*_ is *O*(*mn* ^*K*+1^) [20], where we assume that *h* and *k* are *O*(*n*). Therefore, the time complexity for *σ*_*h*_*j*_,*k*_*j*_,*j*_[*p*, *q*]s is *O*(*m* ^3^ *n* ^*K*+1^), because *p* and *q* are *O*(*m*).

Next, we analyze the time complexity required for computing *D*[*h*, *k*, *i*, *p*, *q*]s. In this computation, we note that the size of table *D*[*h*, *k*, *i*, *p*, *q*] is *O*(*m* ^2^ *n* ^3^). Furthermore, in order to compute the minimum value for each entry in the DP procedure, we need to examine (*H* + 1)(*K* + 1) combinations, which is *O*(1). Hence, the time complexity for *D*[*h*, *k*, *i*, *p*, *q*]s is *O*(*m* ^2^ *n* ^3^).

Finally, we analyze the time complexity required for computing *E*[*h*, *k*, *b*, *q*]s. We note that the size of table *E*[*h*, *k*, *b*, *q*] is *O*(*mn* ^2^), where we assume that *B* is a constant. Since the number of combinations for computing the minimum value using DP is *O*(*mn*) per entry, the computation time required for computing *E*[*h*, *k*, *b*, *q*]s is *O*(*m* ^2^ *n* ^3^). Hence, the total time complexity is

(18) $$\begin{array}{c} O\left(m^{3}n^{K+1}+m^{2}n^{3}\right). \end{array}$$

It is to be noted that if we use *N* time series datasets, each of which consists of *m* points, the time complexity becomes *O*(*Nm* ^3^ *n* ^*K*+1^ + *m* ^2^ *n* ^3^). Although this complexity is not small, it is allowable in practice if *K* ≤ 2 and *n* and *m* are not too large. Indeed, as shown in Section 4.2, DPLSQ-TV works for the completion and inference of time-varying networks with a few tens of genes if *K* = 2.

## 3. Heuristic Method

Although our previous algorithm, DPLSQ-TV, is guaranteed to find an optimal solution in polynomial time, the degree of the polynomial is not low, preventing the method from being applied to the completion of large-scale networks. Therefore, we propose a heuristic algorithm, DPLSQ-HS, to significantly improve the computational efficiency by relaxing the optimality condition. The reason why DPLSQ-TV requires a large amount of CPU time is that the least squares errors are calculated for each node by considering all possible combinations of incoming nodes and taking the minimum value of these. In order to significantly improve the computational efficiency, we introduce an upper limit on the number of combinations of incoming nodes. Although DPLSQ-HS does not guarantee an optimal solution, it allows us to speed up the calculation of the minimum least squares in the case of adding edges. A schematic illustration of least squares computation is given in Section 3.1. The DPLSQ-HS algorithm is described in Section 3.2, and we analyze the time complexity of DPLSQ-HS in Section 3.3.

### 3.1. Schematic Illustrations of DPLSQ-HS

**Figure 3:**
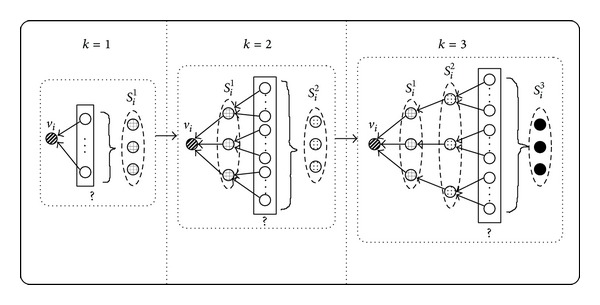
Schematic illustrations for definition of the top *M* combinations. This is an example for the cases of *M* = 3 and *k* ≤ 3. Let *S*_*i*_^*k*^ denote the set of *M* combinations computed at the *k*th step. At the *k*th step, for each combination {*v*_*i*_1__,…, *v*_*i*_*k*−1__} ∈ *S*_*i*_^1^ ∪ *S*_*i*_^2^ ∪⋯∪ *S*_*i*_^*k*−1^, we calculate the least squares error for each *v*_*j*_ such that *j* > *i*_*k*−1_ as a *k*th incoming node to *v*_*i*_.

Although DPLSQ-HS can be applied to the addition and deletion of edges, we consider only additions of edges as modification operations in this subsection. We have developed DPLSQ-HS, which contributes to reducing the time complexity, by imposing restrictions on the number of combinations of incoming nodes to each node. In Figure 3, the diagram indicates that, for each node *v*_*i*_, we maintain *M* combinations of *k* incoming nodes with *M* lowest errors at the *k*th step. Let *S*_*i*_^*k*^ denote the set of *M* combinations computed at the *k*th step. At the *k*th step, for each combination {*v*_*i*_1__,…, *v*_*i*_*k*−1__} ∈ *S*_*i*_^1^ ∪ *S*_*i*_^2^ ∪⋯∪ *S*_*i*_^*k*−1^ where *i*_1_ < *i*_2_ < ⋯<*i*_*k*−1_, we calculate the least squares error for each *v*_*j*_ such that *j* > *i*_*k*−1_ is the *k*th incoming node to *v*_*i*_. The calculated least squares errors are sorted in descending order, the top *M* values are selected, and the corresponding combinations are stored in *S*_*i*_^*k*^.

### 3.2. Algorithm

The following is the description of the algorithm to compute *σ*_*k*,*i*_^+^[*p*, *q*] in DPLSQ-HS, where *σ*_*k*,*i*_^+^[*p*, *q*] does not necessarily mean the minimum value and the meaning of “step” is different from that in Section 3.1.

**Step 1**

For each period [*p*, *q*], repeat Steps 2–6.

**Step 2**

Let *S*_*i*_^0^ = {*∅*} for all *i* = 1,…, *n*.

**Step 3**

For *i* = 1 to *n* do Steps 4–7.

**Step 4**

Repeat Steps 5–7 for node *v*_*i*_ from *k* = 1 to *k* = *K*.

**Step 5**

For each combination {*v*_*i*_1__,…, *v*_*i*_*k*−1__} ∈ *S*_*i*_^1^ ∪ *S*_*i*_^2^ ∪⋯∪ *S*_*i*_^*k*−1^ and each node *v*_*j*_ such that *j* > *i*_*k*−1_ (*j* > 0 if *k* = 1), calculate the least squares error for the *k* edge set {(*v*_*i*_1__, *v*_*i*_),…, (*v*_*i*_*k*−1__, *v*_*i*_), (*v*_*j*_, *v*_*i*_)}.

**Step 6**

Sort the obtained least squares errors in descending order and select the top *M* combinations, which are stored in *S*_*i*_^*k*^.

**Step 7**

Let *σ*_*k*,*i*_^+^[*p*, *q*] be the minimum least squares error among these top *M* combinations.

The other parts of the algorithm are the same as in DPLSQ-TV.

### 3.3. Time Complexity Analysis

In this subsection, we analyze the time complexity of DPLSQ-HS. Since DPLSQ-HS can be applied to additions and deletions of edges, we consider the time complexity of completion for adding and deleting edges.

In our proposed method, we assume that the numbers of adding and deleting edges in a given network are, respectively, bounded by constants *K* and *H*. In this case, the time complexity for least squares fitting can be estimated as *O*(*m*).

As for the time complexity of computing *σ*_*h*_*j*_,*k*_*j*_,*j*_[*p*, *q*], we assume that the addition of edges is operated only in the case of adding edges to the nodes with respect to the top *M* of the sorted list. Therefore, the number of combinations of addition of *k*_*j*_ edges, which is bounded by a constant *K*, is *O*(*M* ^*K*^). It is well known that the sorting of *n* data can be done in *O*(*n*log⁡*n*) time. Based on such an assumption, the total time required for the computation of *σ*_*h*_*j*_,*k*_*j*_,*j*_[*p*, *q*] is *O*(*mn*log⁡*n*) [20], since the *O*(*M* ^*K*^) factor can be regarded as a constant. Therefore, the time complexity for *σ*_*h*_*j*_,*k*_*j*_,*j*_[*p*, *q*] is *O*(*m* ^3^ *n*log⁡*n*), because *p* and *q* are *O*(*m*).

Furthermore, for the time complexity required for computing *D*[*h*, *k*, *i*, *p*, *q*]s and *E*[*h*, *k*, *b*, *q*]s, the calculation process is the same as that in DPLSQ-TV. Therefore, the computation time for both *D*[*h*, *k*, *i*, *p*, *q*]s and *E*[*h*, *k*, *b*, *q*]s are *O*(*m* ^2^ *n* ^3^) as described in Section 2.4. Hence, the total time complexity of DPLSQ-HS is

(19) $$\begin{array}{c} O\left(m^{3}n\log n+m^{2}n^{3}\right). \end{array}$$

If we use *N* time series datasets, each of which consists of *m* points, the time complexity becomes *O*(*Nm* ^3^log⁡*n* + *m* ^2^ *n* ^3^). DPLSQ-HS requires less time complexity than DPLSQ-TV, because *O*(*m* ^3^ *n*log⁡*n*) is much smaller than *O*(*m* ^3^ *n* ^*K*+1^). Indeed, as shown in Section 4.2, DPLSQ-HS is much faster than DPLSQ-TV in practice.

## 4. Results

We performed computational experiments using both artificial data and real data. All experiments were performed on a PC with an Intel Core(TM)2 Quad CPU (3.0 GHz). We employed the liblsq library (http://www2.nict.go.jp/aeri/sts/stmg/K5/VSSP/install_lsq.html) for the least squares fitting method.

### 4.1. Completion Using Artificial Data

**Figure 4:**
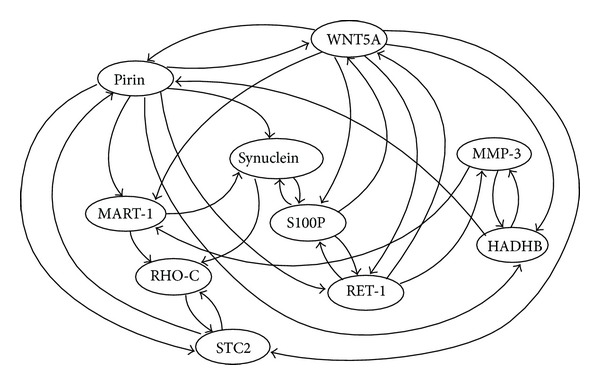
Structure of WNT5A network [26].

In order to evaluate the potential effectiveness of DPLSQ-TV and DPLSQ-HS, we begin with network completion for time-varying networks using artificial data. We demonstrate that our proposed methods can determine change time points quite accurately when the network structure changes. We employed the structure of the real biological network WNT5A (Figure 4) [26] as the initial network *G* and those of three different networks *G*_1_, *G*_2_, and *G*_3_ generated by randomly adding and deleting edges from the initial network. In this method, for each node *v*_*i*_ with *h* input nodes, we considered the following model:

(20) xi(t+1) =xi(t)+Δt(a0i+∑j=1hajixij+∑j<kaj,kixij(t)xik(t)+biω),

where *a*_*j*_^*i*^s and *a*_*j*,*k*_^*i*^s are constants selected uniformly at random from [−0.5,0.5] and [−0.05,0.05], respectively. The reason why the domain of *a*_*j*,*k*_^*i*^s is smaller than that for *a*_*j*_^*i*^s is that nonlinear terms are not considered as strong as linear terms. It should also be noted that *b*_*i*_ *ω* is a stochastic term, where *b*_*i*_ is a constant (we used *b*_*i*_ = 0.05) and *ω* is random noise taken uniformly at random from [−1,1]. For the artificial generation of the observed data *y*_*i*_(*t*), we used

(21) $$\begin{array}{c} y_{i}\left(t\right)=x_{i}\left(t\right)+o^{i}\epsilon , \end{array}$$

where *o* ^*i*^ is a constant denoting the level of observation errors and *ϵ* is random noise taken uniformly at random from [0.05, −0.05].

As for the time series data, we generated an original dataset with 30 time points including two change points *c*_1_ = 10, *c*_2_ = 20, which is generated by merging three datasets for *G*_1_, *G*_2_, and *G*_3_. Since the use of time series data beginning from only one set of initial values easily resulted in numerical calculation errors, we generated additional time series data beginning from 200 sets of initial values that were obtained by slightly perturbing the original data. Under the above model, we conducted computational experiments by DPLSQ-TV in which the initial network *G* was modified by randomly adding *k*_0_ edges and deleting *h*_0_ edges per node, resulting in *G*_1_, *G*_2_, and *G*_3_; additionally, we also conducted DPLSQ-HS experiments in which the initial network *G* was modified by randomly adding *k*_0_ edges per node, using the default values of *k*_0_ = *h*_0_ = 1. We evaluated the performance of this method by measuring the accuracy of modified edges, the time point errors for time intervals, and the computational time for completion (CPU time). Furthermore, in order to examine how CPU time changes as the size of the network grows, we generated networks with 20 genes, 30 genes, and 40 genes by making 2, 3, and 4 copies of the original networks. We took the average time point errors, accuracies, and CPU time over 10 random modifications with several *o* ^*i*^s. In addition, we performed computational experiments on DPLSQ-TV and DPLSQ-HS using 60 genes, where additional time series data beginning from 100 sets (in place of 200 sets) of initial values were used, and *G*_1_, *G*_2_, and *G*_3_ were obtained by addition and deletion of edges. However, DPLSQ-TV took too long time (more than 1000 sec. per execution) and thus the result could not be included in Table 1.

The accuracy is defined as follows:

(22) $$\begin{array}{c} \frac{h+k+\sum_{i=0}^{B}\left(\left|E_{i}\cap E_{i}^{\prime }\right|-\left|E_{i}\right|\right)}{h+k}, \end{array}$$

where *E*_*i*_ and *E*_*i*_′(*i* = 0,1,…, *B*) are, respectively, the sets of edges in the original network and the completed network in each time interval. This value is 1 if all the added and deleted edges are correct and 0 if none of the added and deleted edges are correct. If we regard a correctly (resp., incorrectly) added or deleted edge as a true (resp., false) positive, ∑_*i*=0_^*B*^(|*E*_*i*_ | −|*E*_*i*_∩*E*_*i*_′|) corresponds to the number of false positives and *h* + *k* + ∑_*i*=0_^*B*^(|*E*_*i*_∩*E*_*i*_′ | −|*E*_*i*_|) corresponds to the number of true positives. The time point error is the average difference between the original and estimated values for change time points and is defined as

(23) $$\begin{array}{c} \frac{1}{B}\overset{B}{\underset{i=1}{\sum }}\left|c_{i}-c_{i}^{\prime }\right|, \end{array}$$

where *c*_*i*_′  (*i* = 1,2,…, *B*) are the estimated change points. As for the computation time, we show the average CPU time.

The results of the two methods are shown in Table 1. It can be seen from this table that the change time point errors are quite small regardless of the size of the network with a low level of observation errors. In addition, it is also seen that the time point errors with DPLSQ-TV are close to those with DPLSQ-HS with the exception of high levels of observation errors. We observe that CPU time using DPLSQ-TV increases rapidly as the size of the network grows. On the other hand, CPU time by DPLSQ-HS increases gradually as the size of the network grows. It is also observed that the DPLSQ-HS algorithm is about 4 times faster than the DPLSQ-TV algorithm in case of 40 genes, while maintaining good accuracy. Hence, these results suggest that DPLSQ-TV and DPLSQ-HS can correctly identify the change time points if the error levels are not very large and that it can complete the initial network by modifying the edges with relatively good accuracy if the observation error is small.

It is also observed that DPLSQ-HS worked reasonably fast even for *n* = 60, although DPLSQ-TV took more than 1000 seconds per execution and thus the result could not be included in Table 1. However, the accuracy on DPLSQ-HS became around 0.4 even if the observation error level was low (i.e., *o* ^*i*^ = 0.1). Therefore, the applicability of DPLSQ-HS is also limited in terms of the accuracy, although it may still be useful for networks with *n* = 60 if the purpose is to identify change time points.

Since DPLSQ-HS is a heuristic method, the results may be greatly influenced by data. Therefore, we evaluated the stability of DPLSQ-HS by comparing the variance of the accuracy with that for DPLSQ-TV, where *n* = 20. The variances for DPLSQ-TV were 0.00602 and 0.00446 for *o* ^*i*^ = 0.3 and *o* ^*i*^ = 0.5, respectively. The variances for DPLSQ-HS were 0.01188 and 0.00732 for *o* ^*i*^ = 0.3 and *o* ^*i*^ = 0.5, respectively. This result suggests that DPLSQ-HS is less stable than DPLSQ-TV. However, the variances of DPLSQ-HS were less than twice those of DPLSQ-TV. Therefore, this result also suggests that DPLSQ-HS has some stability.

In order to examine the effect of the number of change points *B* and the maximum number of added and deleted edges per nodes *K* and *H* on the least squares error, we performed computational experiments with varying these parameters (one experiment per parameter). Then, the resulting least squares errors (i.e., *E*[⋯]s) for DPLSQ-TV are 5.495, 7.016, 7.875, 3.886, and 3.799 for (*B*, *K*, *H*) = (2,1, 1), (3,1, 1), (4,1, 1), (2,2, 2), and (2,4, 2), respectively. It is seen that use of larger *K*, *H* resulted in smaller least squares errors. It is reasonable that more parameters resulted in better least squares fitting. However, use of larger *B* did not result in smaller least squares errors. It may be because addition of unnecessary change points increases the error if an enough number of edges are not added. It is to be noted that although the least squares errors are reduced, use of larger *K*, *H* is not always appropriate because it needs much longer CPU time and may cause overfitting.

**Figure 5:**
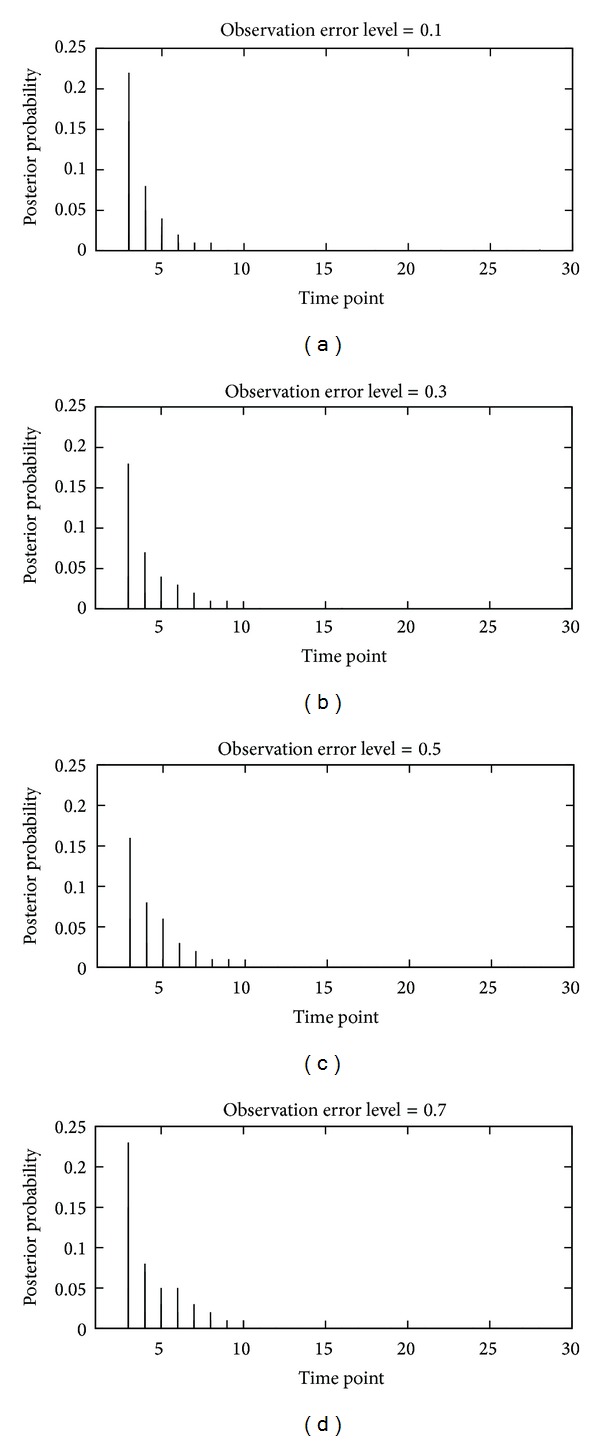
Results on inference of change point using ARTIVA algorithm with synthetic data.

We also compared our results with those obtained by the ARTIVA algorithm [15]. It is to be noted that most of the other tools for the inference of time-varying networks are unavailable. This model is based on a combination of DBN and RJMCMC sampling, where RJMCMC is used for approximating the posterior distribution and DBN is used for inferring simultaneously the change points and resulting network structures. We applied ARTIVA to the synthetic datasets that were generated in the same way as for our proposed methods. We used the default parameter settings for ARTIVA and evaluated the results by inferring the change points. As the result of the comparative experiment, there are two change time points in the synthetic datasets, but ARTIVA can only infer one change point regardless of the observation error level, as shown in Figure 5, where ARTIVA does not uniquely determine change points but output probabilities of change points.

### 4.2. Inference Using Real Data

We examined two types of proposed methods for the inference of change time points using gene expression microarray data and also compared our results with those obtained using the ARTIVA algorithm. We applied our methods to two real gene expression datasets, measured during the life cycle of* D. melanogaster* and the cell cycle of* S. cerevisiae*.

**Figure 6:**
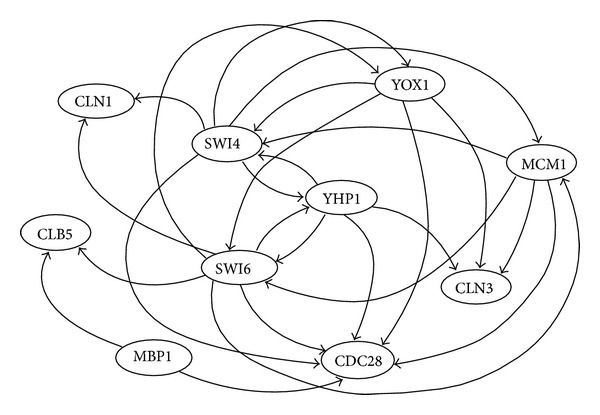
Structure of part of the yeast cell cycle network.

The first microarray dataset is the gene expression time series collected by Spellman et al. [28]. We employed part of the cell cycle network of* S. cerevisiae* extracted from the KEGG database [29] shown in Figure 6. As for time series data, we combined and employed four sets of time series data (alpha, cdc15, cdc28, and elu) in [28] that were obtained in four different experiments. We adopted the datasets of 10 genes with 71 time points including three change time points. Since there were several expression values that were far from the average in the cdc15 dataset, these values were discarded. As a result, the alpha, cdc15, cdc28, and elu datasets consist of 18, 23, 17, and 13 time points of gene expression data, respectively.

The second microarray dataset is the gene expression time series from experiments by Arbeitman et al. [30]. This data set includes the expression levels of 4028 genes with 67 time points spanning four distinct stages: embryonic (31 time points), larval (10 time points), pupal (18 time points), and adulthood (8 time points) in the* D. melanogaster* life cycle. We used the expression datasets of 30 genes selected from this microarray data with 67 time points, which include three change time points.

In this computational analysis, with regard to applying the two different types of microarray datasets, we generated 200 datasets that were obtained by slightly perturbing the original data in order to avoid numerical calculation errors. Since the correct time-varying networks are not known, we only evaluated the time point errors and the average CPU time, where *K* = 3 and *H* = 0 were used with the* S. cerevisiae* dataset and *K* = 2 and *H* = 0 were used with the* D. melanogaster* dataset.

The results are shown in Tables 2 and 3. *c*_*i*_s are the values of the change point in the original data and *c*_*i*_′s are the estimated values. In the experimental analysis with* S. cerevisiae* data, as for the change time points, there seems to be almost no difference between the results of DPLSQ-TV and DPLSQ-HS, which can correctly identify the time points where the network topology changes. It is also observed from Table 2 that the CPU time required for DPLSQ-HS is about 15 times faster than that needed for DPLSQ-TV. In the experiments using data from* D. melanogaster*, it is seen from Table 3 that both methods can determine exactly the same three change points. At first glance, readers may think that the errors are large at all change point positions. However, both methods could precisely identify two time points when topology of the network changes, excluding the case of *i* = 3. From the point of view of computational time, DPLSQ-HS performs significantly better than DPLSQ-TV; DPLSQ-HS runs about 46 times faster than DPLSQ-TV. Therefore, DPLSQ-HS allows us to significantly decrease the computational time. These results suggest that, in many cases, we can expect DPLSQ-HS to find a near-optimal solution, at least for change time points, while also speeding up the calculation.

Furthermore, for the ARTIVA analysis, we employed both the above-mentioned* S. cerevisiae* and* D. melanogaster* microarray datasets, which consist of 71 measurements of 10 genes and 67 measurements of 30 genes, respectively, and tried to identify the change time points. Computational experiments on ARTIVA were performed under the same computational environment as that used in our methods.

The results from the yeast microarray data are shown in Table 2. There are three change time points, as described in this table. It is seen from this table that two of them, 24 and 60, can be determined precisely by ARTIVA, but the third is not. In contrast, our proposed methods demonstrate good performance for inferring the change points at which the network topology changes. Lèbre et al. [15] demonstrated the number of identified change points with* D. melanogaster* data using the ARTIVA algorithm. According to this validation, it has been observed that the time intervals 18-19, 31–33, 41–43, and 59–61 contain more than 40% of all change points. In order to compare with the ARTIVA results, we attempted to identify four change points using our proposed methods. The results of the comparative experiment using* D. melanogaster* microarray data are shown in Table 4. *c*_*i*_s are three change time points in original data. Although DPLSQ-HS identified change time points similar to those identified by ARTIVA, the results of ARTIVA appear to be slightly better. This suggests that the ARTIVA algorithm shows slightly better performance with respect to the inference of change points than our proposed methods. However, ARTIVA does not determine change time positions but determines time intervals at which the network topology might change. Therefore, DPLSQ-HS is more suited for identifying change time positions at all-time points. (Since the comparative experiment by DPLSQ-TV did not finish within 3 weeks, the results of DPLSQ-TV are not given in Table 4.)

## 5. Conclusion

In this paper, we have proposed two novel network completion methods for time-varying networks by extending our previous method, DPLSQ [20]. In order to identify the change time points and sets of modified edges in network completion, we developed two different types of double DP algorithms. The first algorithm, DPLSQ-TV, is intended to complete and precisely infer time-varying networks. Although DPLSQ-TV allows us to guarantee the optimality of its solution, it requires a large amount of computational time as the size of the network grows.

To improve the computational efficiency of DPLSQ-TV, we developed an effective heuristic method, DPLSQ-HS, by speeding up the calculation of the minimum least squares error by posing restrictions to the number of combinations of incoming nodes. We showed that each of these two methods works in polynomial time if the maximum indegree is bounded by a constant.

The results of computational experiments reveal that the two proposed methods can identify change time points rather accurately and can infer edges to be deleted and added with good accuracy. DPLSQ-TV provided a wide range of applications, not only in network completion but also in network inference, with good accuracy. Additionally, DPLSQ-HS allowed us to identify change time points rather precisely, while reducing the computational time for both synthetic data and microarray data. This result suggests that, in many cases, DPLSQ-HS can be expected to find near-optimal solutions, while speeding up the calculation.

Although DPLSQ-HS is much faster than DPLSQ-TV, it has a drawback: the accuracy and time point error were worse than those by DPLSQ-TV, especially, when the observation error level was large. Therefore, we need to improve the accuracy of DPLSQ-HS without significantly undermining its efficiency. In our experiments, we specified the number of change time points and the number of edges to be added and deleted. In real use, we may examine several values and select the best one (e.g., the values with the minimum least squares errors). However, as discussed in Section 4.1, it may lead to overfitting. In order to avoid overfitting, use of AIC (Akaike's Information Criterion) or other information criteria is useful as demonstrated in [27] for network inference. However, since network completion is more complex than network inference, the method in [27] cannot be directly applied. Therefore, incorporation of an appropriate information criterion into network completion is important future work. Another issue to be tackled is to take into account the relationship between *G*_*i*_ and *G*_*i*+1_. Although *G*_*i*_ and *G*_*i*+1_ are inferred independently from the original network *G* by the proposed method, there should be some strong relationship between them. Therefore, such an extension is also important future work.

## Figures and Tables

**Table:** (a) Using DPLSQ-TV

		Observation error level			
		0.1	0.3	0.5	0.7
*n* = 10	Time points error	0.00	0.00	0.00	1.60
	Accuracy	0.830	0.685	0.554	0.433
	CPU time (sec.)	53.974	58.574	50.499	61.189

*n* = 20	Time points error	0.00	0.00	0.00	2.10
	Accuracy	0.781	0.637	0.541	0.299
	CPU time (sec.)	75.898	85.391	142.903	124.400

*n* = 30	Time points error	0.00	0.00	0.35	4.00
	Accuracy	0.539	0.524	0.418	0.310
	CPU time (sec.)	264.190	244.480	234.377	276.081

*n* = 40	Time points error	0.00	0.00	0.35	3.50
	Accuracy	0.585	0.498	0.458	0.241
	CPU time (sec.)	342.065	333.398	317.420	337.274

**Table:** (b) Using DPLSQ-HS

		Observation error level			
		0.1	0.3	0.5	0.7
*n* = 10	Time points error	0.00	0.00	0.00	6.85
	Accuracy	0.627	0.600	0.533	0.307
	CPU time (sec.)	32.783	32.030	35.594	30.765

*n* = 20	Time points error	0.00	0.00	0.00	8.70
	Accuracy	0.488	0.473	0.413	0.153
	CPU time (sec.)	52.129	51.348	56.723	44.699

*n* = 30	time points error	0.00	0.00	4.25	8.80
	Accuracy	0.469	0.386	0.286	0.097
	CPU time (sec.)	76.852	86.844	76.313	74.653

*n* = 40	Time points error	0.00	0.00	0.85	8.65
	Accuracy	0.510	0.413	0.308	0.093
	CPU time (sec.)	76.360	98.398	97.657	102.813

*n* = 60	Time points error	0.00	0.00	0.00	0.75
	Accuracy	0.411	0.375	0.382	0.355
	CPU time (sec.)	371.635	395.596	372.192	391.110

**Table 2:** Result on inference of change points in *S. cerevisiae* data.

	*c*_*i*_ (correct answer)	*c*_*i*_′ (DPLSQ-TV)	*c*_*i*_′ (DPLSQ-HS)	ARTIVA
*i* = 1	25	23	23	24
*i* = 2	40	40	40	—
*i* = 3	56	58	58	60
CPU time (sec.)		14147.18	908.80	—

**Table 3:** Result on inference of change points in *D. melanogaster* data.

	*c*_*i*_ (correct answer)	*c*_*i*_′ (DPLSQ-TV)	*c*_*i*_′ (DPLSQ-HS)
*i* = 1	31	19	19
*i* = 2	41	31	31
*i* = 3	60	42	42
CPU time (sec.)		121560.82	2620.96

**Table 4:** Comparative experiment for inference of change points.

	*c*_*i*_ (correct answer)	*c*_*i*_′ (DPLSQ-HS)	ARTIVA
*i* = 1	—	19	18-19
*i* = 2	31	31	31–33
*i* = 3	40	42	41–43
*i* = 4	60	55	59–61
CPU time (sec.)		2606.40	—
